# Examining the interplay between sdLDL, resistin, and BMI

**DOI:** 10.1038/s41598-025-26772-w

**Published:** 2025-11-28

**Authors:** Fauzia Ashfaq, Mohammad Idreesh Khan, Abdulrahman A. Alsayegh, Mohammed Bajahzer, Mohammed Abdullah Jeraiby, Fahad Saad Alhodieb, Fahmida Khatoon, Saleh Lafi Alshammari, Mirza Masroor Ali Beg

**Affiliations:** 1 Department of Clinical Nutrition, College of Nursing and Health Sciences, Jazan University, Jazan, 45142 Saudi Arabia; 2 Department of Basic Health Sciences, College of Applied Medical Sciences, Qassim University, P.O. Box 6666, Buraydah, 51452 Saudi Arabia; 3 Department of Basic Medical Science, College of Medicine, Jazan University, Jazan, 45142, Saudi Arabia; 4 Biochemistry Department, College of Medicine, University of Hail, Hail, Saudi Arabia; 5 Prince Sultan Cardiac Center & Health Services Directorate, P.O. Box 65434, Riyadh, Saudi Arabia; 6 Faculty of Medicine, Alatoo International University, Bishkek, Kyrgyzstan

**Keywords:** Small dense LDL, Resistin, Body mass index, Obesity, Prognostic marker, Biochemistry, Biomarkers, Diseases, Endocrinology, Health care, Medical research

## Abstract

Obesity is a major risk factor for many chronic diseases and is becoming a serious global health problem. Poor health outcomes are closely linked to body mass index (BMI). In obesity, changes in small dense low-density lipoprotein (sdLDL) may increase its ability to penetrate the endothelium, leading to atherosclerosis. Obesity also affects the metabolic and secretory functions of many tissues, which may raise serum resistin levels. This study included 300 participants with different BMI levels. We measured sdLDL and resistin in all participants. HbA1c was tested using whole blood in EDTA tubes. Serum from thawed samples was used to measure lipid parameters (triglycerides, cholesterol, HDL, LDL, VLDL, sdLDL) and resistin levels were analysed using ELISA. Obese participants had significantly higher HbA1c (*p* = 0.0004), LDL (*p* < 0.0001), triglycerides (*p* < 0.0001), cholesterol (*p* < 0.0001), and VLDL (*p* < 0.0001) compared to overweight and normal BMI groups, except for HDL, which was not significantly different. Smokers and participants with hypertension showed higher sdLDL (*p* = 0.03, *p* = 0.0001) and resistin levels (*p* = 0.03, *p* < 0.0001). Overweight participants had sdLDL levels of 21.95 mg/dl, while obese participants had 25.76 mg/dl, both significantly higher than the normal BMI group (*p* < 0.0001 for both). Resistin levels were also higher in overweight (1003 pg/mL) and obese participants (1355 pg/mL) compared to the normal BMI group (*p* < 0.0001 for *p* < 0.0001 ). SdLDL and resistin showed a positive correlation with each other and were significantly associated with BMI, systolic blood pressure, triglycerides, cholesterol, VLDL, and LDL, but negatively associated with HDL. ROC analysis indicated that sdLDL (cutoff: 18.55 mg/dl) and resistin (cutoff: 750 pg/mL) could serve as prognostic markers for overweight and obesity. Additionally, participants with normal BMI had resistin levels of 389.6 pg/mL, overweight participants had 300.6 pg/mL (*p* < 0.0001), and obese participants had 291.0 pg/mL (*p* < 0.0001). This study suggests that resistin and sdLDL levels are the primary cause of dyslipidemia and metabolic dysregulation in obesity. Both biomarkers are strong predictors of a higher BMI category, with resistin being a universal risk factor and sdLDL showing a male-specific correlation. Additionally, their high predictive accuracy confirms the usefulness of sdLDL and resistin as biomarkers for early risk assessment and shows how they can guide early intervention strategies for metabolic and cardiovascular risk linked to obesity.

## Introduction

Obesity is a well-established risk factor for numerous chronic conditions, including type 2 diabetes mellitus (T2DM), cardiovascular disease (CVD), atherosclerosis, and all-cause mortality^1^. Small dense LDL cholesterol (sdLDL-c) has been strongly linked to these metabolic disorders and is now considered an emerging risk factor for CVD and T2DM. The “proatherogenic lipoprotein phenotype” characterized by elevated triglycerides (TG) and low HDL is common in T2DM and metabolic syndrome (MetS)^2^ and is often accompanied by high circulating sdLDL levels, particularly in obesity and systemic inflammation^3^. Inflammatory processes can directly alter lipid metabolism, increasing TG, very low-density lipoproteins (VLDL), and ultimately sdLDL concentrations^4,5^.

Obesity promotes an atherogenic lipid profile and exacerbates cardiometabolic risk factors^6^. Lower body weight is generally associated with improved lipid levels and better disease outcomes, highlighting the importance of early interventions to reduce excess adiposity and atherogenic cholesterol levels. SdLDL particles are also closely linked to insulin resistance, with their atherogenicity attributed to rapid arterial wall penetration and increased susceptibility to oxidation^7^. South Asians are particularly vulnerable to T2DM and CVD due to central fat distribution, even at lower BMI thresholds^8^, and overweight individuals often exhibit higher TG, LDL, and greater LDL oxidation sensitivity^9^. Measuring LDL subclasses, especially sdLDL, can enhance cardiovascular risk assessment in MetS^10–12^, and sdLDL is a key marker of metabolically unhealthy obesity^13^. This shift toward more atherogenic particles has also been observed in obese children, particularly those with hypertension^14^.

Resistin, an adipokine produced during adipogenesis, is linked to insulin resistance in obesity and diabetes^15^. Higher serum resistin levels are consistently reported in obese individuals compared to lean individuals^16–19^ and are correlated with visceral fat and BMI^17^. Resistin secretion is interconnected with obesity, insulin resistance, and adiposity^20^. Experimental models show that elevated resistin levels in obesity can be reduced with diabetes treatment, and lowering resistin improves insulin sensitivity and glucose control^20^.

Obesity is a significant cause of cardiometabolic diseases in Saudi Arabia, despite the fact that traditional clinical evaluation usually overlooks sophisticated lipid markers and adipokines that could allow for an earlier risk diagnosis. While sdLDL is highly atherogenic due to its small size, increased artery wall penetration, and oxidative vulnerability, resistin plays a role in insulin resistance and systemic inflammation. Understanding how these two indicators interact across different BMI ranges may aid in the pathophysiology of obesity-related problems. By evaluating sdLDL and resistin together in a Saudi Arabian population, this study seeks to identify new predictive biomarkers that could enhance the assessment of cardiovascular and metabolic risk. For high-risk individuals, this will enable earlier and more targeted interventions. Given these associations, the present study aimed to examine alterations in sdLDL and serum resistin levels, as well as their interrelationship across different BMI categories in the Saudi Arabian population.

## Materials and methods

### Subjects and study design

**Fig. 1:**
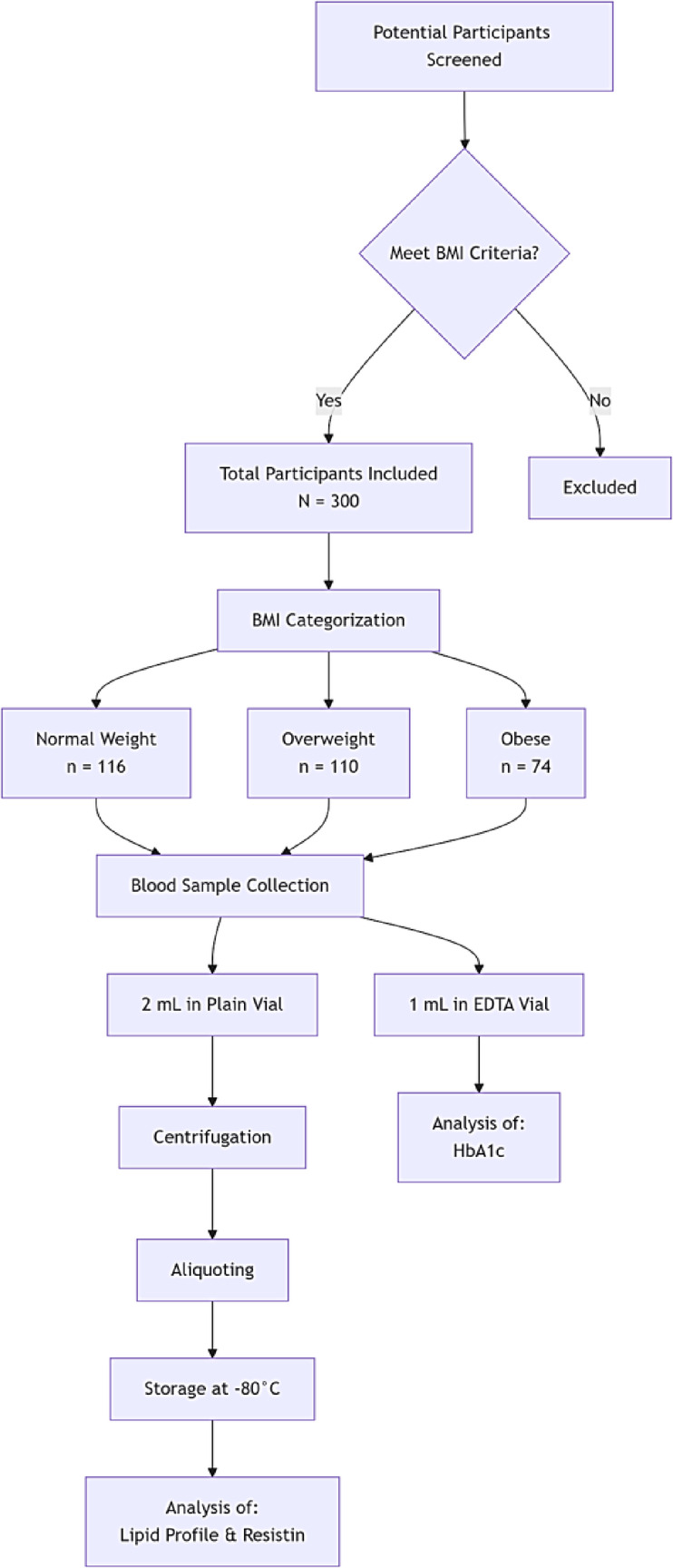
Flow chart of study design, participant selection, experimentation, and analysis.

The present cross-sectional observational study included 300 participants (male and female), categorized by BMI as normal weight (18.5–24.9 kg/m², *n* = 116, 38.7%), overweight (25–29.9 kg/m², *n* = 110, 36.7%), and obese (≥ 30 kg/m², *n* = 74, 24.6%). Participants were not age- or sex-matched, which is acknowledged as a study limitation. To reduce confounding and focus on BMI-related differences, individuals with chronic diseases, including cardiovascular disease, type 2 diabetes, kidney or liver disorders, and thyroid dysfunction, were excluded. All procedures adhered to the Declaration of Helsinki, and written informed consent was obtained from all participants before sample collection and for publication of study data. The University of Hail’s Research Ethics Committee in Saudi Arabia gave its approval to the study (Approval no: H-2023-014) was conducted between 2023 and 2024. Before the study started, all participants gave their written informed consent (Fig. 1).

### Sample collection and processing

Peripheral blood samples were collected after BMI-based screening. One milliliter of blood was collected in EDTA vials for HbA1c measurement, and 2 mL was collected in plain vials for serum separation. Plain vial samples were centrifuged at 1500 rpm for 10 min to separate serum, which was aliquoted and stored at -80 °C until analysis.

### Biochemical analysis

Lipid profile parameters including triglycerides (TG), total cholesterol, HDL, LDL, VLDL, and small dense LDL (sdLDL) were measured from thawed serum using commercial kits (Randox Laboratories Limited, Antrim, United Kingdom). HbA1c was determined from EDTA blood samples using standard laboratory protocols.

### ELISA for resistin

Serum resistin levels were measured using a Human Resistin ELISA Kit (ab183364, Abcam, San Francisco, CA, US) following the manufacturer’s instructions. Thawed serum samples were centrifuged and analyzed in 96-well plates for all participants across BMI categories.

### Statistical analysis

Microsoft Excel was used to record the data, and SPSS version 21 and GraphPad Prism version 6.05. The Kolmogorov-Smirnov test was used to determine whether the data were normal. Non-parametric tests were employed because a number of variables did not satisfy the normality assumptions, based on the skewness of output or data the different statistical test were used. The Mann-Whitney U-test (Resistin (pg/mL) was used to compare quantitative data between two groups, and the Kruskal-Wallis (Resistin (pg/mL) test was used to compare quantitative data between more than two groups. However, for the parametric T test (sdLDL) for 2 groups and ANOVA (HbA1c, lipid profile, (sdLDL) for more than 2 groups were used. Spearman’s correlation and multivariate logistic regression analysis was used to evaluate relationships between variables. Differences were deemed statistically significant at *p* < 0.05, and results are presented as mean ± standard deviation.

## Results

### Demographic of study participants

Demographic and general characteristic of study participants were depicted (Table 1). Present research work had total 300 study participants among them 85.7% were males and 14.3% were females. Participants with *≤* 40 age group were 61.4% and > 40 years age group were 38.6%.

**Table 1:** General demographic details of the study’s participants

Variables	Participants (%)
Total no.	300 (100)
Gender	
Males	257 (85.7)
Females	47 (14.3)
Age (Years)	
*≤* 40 years	184 (61.4)
> 40 years	116 (38.6)
Smoking	
Yes	105 (35)
No	195 (65)
Hypertension	
Yes	71 (23.7)
No	229 (76.3)
Physical activity	
Yes	136 (45.3)
No	164 (54.7)
Body mass index kg/m^2^	
Normal	116 (38.7)
Overweight	110 (36.7)
Obesity	74 (24.6)

### Body mass index and biochemical parameters

Glycemic and lipid parameters were assessed in participants with normal BMI, overweight, and obesity (Table 2). From 5.07 ± 0.63 in normal-weight participants to 5.35 ± 0.66 in overweight and 5.42 ± 0.65 in obese participants, the mean HbA1c (%) gradually Rose with BMI (*p* = 0.0004). Analysis of lipid profiles revealed notable changes as BMI increased. The mean HDL dropped from 44.72 ± 12.82 mg/dl in people with a normal BMI to 40.42 ± 12.93 mg/dl in people who were overweight and 34.50 ± 6.91 mg/dl in people who were obese (*p* < 0.0001). On the other hand, compared to normal BMI (108.9 ± 19.40 mg/dl, *p* < 0.0001), LDL levels were significantly higher in overweight people (187.8 ± 30.60 mg/dl) and obese people (195.3 ± 28.62 mg/dl). BMI also caused a significant increase in triglycerides (TG), which were 164.8 ± 18.02 mg/dl (normal), 216.9 ± 32.74 mg/dl (overweight), and 225.6 ± 39.76 mg/dl (obese, *p* < 0.0001). Participants with normal BMI had total cholesterol of 185.8 ± 32.95 mg/dl, while those with overweight and obesity had total cholesterol of 233.2 ± 25.30 mg/dl and 249.1 ± 7.38 mg/dl, respectively (*p* < 0.0001). Overweight (32.26 ± 4.51 mg/dl) and obese (36.92 ± 4.70 mg/dl) groups had significantly higher VLDL levels than those with normal BMI (24.13 ± 5.13 mg/dl, *p* < 0.0001). Higher glycemic and atherogenic lipid levels were generally linked to rising BMI, with notable variations observed across all parameters.

**Table 2:** Clinical and biochemical parameters between healthy controls, overweight participants, and obese participants.

Variables	Normal BMI (mean *±* SD)	Overweight (mean *±* SD)	Obese (mean *±* SD)	*p* value
HbA1c (%)	5.07 *±* 0.63	5.35 *±* 0.66	5.42 *±* 0.65	0.0004
HDL (mg/dl)	44.72 *±* 12.82	40.42 *±* 12.93	34.50 *±* 6.91	< 0.0001
LDL (mg/dl)	108.9 *±* 19.40	187.8 *±* 30.60	195.3 *±* 28.62	< 0.0001
TG (mg/dl)	164.8 *±* 18.02	216.9 *±* 32.74	225.6 *±* 39.76	< 0.0001
Cholesterol (mg/dl)	185.8 *±* 32.95	233.2 *±* 25.30	249.1 *±* 7.38	< 0.0001
VLDL (mg/dl)	24.13 *±* 5.13	32.26 *±* 4.51	36.92 *±* 4.70	< 0.0001
VLDL (TG/5) (mg/dl)	32.96 + 3.60	43.40 + 6.54	45.11 + 7.5	< 0.0001

### Comparison of SdLDL with different variables

The distribution of sdLDL levels was evaluated according to gender, age, smoking status, hypertension, and physical activity (Table 3). Participants who smoked had significantly higher sdLDL levels (21.33 ± 4.45 mg/dl) compared to non-smokers (19.92 ± 5.67 mg/dl, *p* = 0.03). Similarly, individuals with hypertension showed elevated sdLDL (22.53 ± 3.73 mg/dl) relative to those without hypertension (19.73 ± 5.56 mg/dl, *p* = 0.0001). Overall, smoking and hypertension were associated with significantly higher sdLDL levels, while gender, age, and physical activity showed no significant effect.

**Table 3:** Comparison of SdLDL level with different variables (gender, age, smoking habit, hypertension and physical activity

Variables	sdLDL (mg/dl) (mean *±* SD)	*p* value
Gender		
Males	20.47 *±* 5.21	0.81
Females	20.11 *±* 5.91	
Age (Years)		
*≤* 40	20.64 *±* 5.04	0.84
> 40	20.76 *±* 5.07	
Smoking		
Yes	21.33 *±* 4.45	0.03
No	19.92 *±* 5.67	
Hypertension		
Yes	22.53 *±* 3.73	0.0001
No	19.73 *±* 5.56	
Physical activity		
Yes	20.07 *±* 5.50	0.29
No	20.84 *±* 5.06	

### Comparison of resistin with different variables

Level of resistin was compared with different parameters and clinical outcome of study participants (Table 4) and observed to have statistically significant difference with smoking and hypertension parameters. Smokers had significantly higher resistin levels (1047 ± 375.2 pg/mL) compared with non-smokers (932.2 ± 378.8 pg/mL, *p* = 0.01). Likewise, participants with hypertension exhibited markedly elevated resistin concentrations (1136 ± 364.6 pg/mL) relative to normotensive individuals (921.5 ± 372.2 pg/mL, *p* < 0.0001). Overall, higher resistin concentrations were significantly associated with smoking and hypertension, but not with gender, age, or physical activity.

**Table 4:** Comparison of resistin level with different variables (gender, age, smoking habit, hypertension and physical activity

Variables	Resistin (pg/mL) (mean *±* SD)	*p* value
Gender		
Males	982.7 *±* 374	0.21
Females	910.1 *±* 418.9	
Age (Years)		
*≤* 40	979.9 *±* 383.8	0.71
> 40	960.5 *±* 377.6	
Smoking		
Yes	1047 *±* 375.2	0.01
No	932.2 *±* 378.8	
Hypertension		
Yes	1136 *±* 364.6	< 0.0001
No	921.5 *±* 372.2	
Physical activity		
Yes	989.1 *±* 352.2	0.40
No	958.3 *±* 403.7	

### Association of sdLDL, and resistin with BMI

**Fig. 2:**
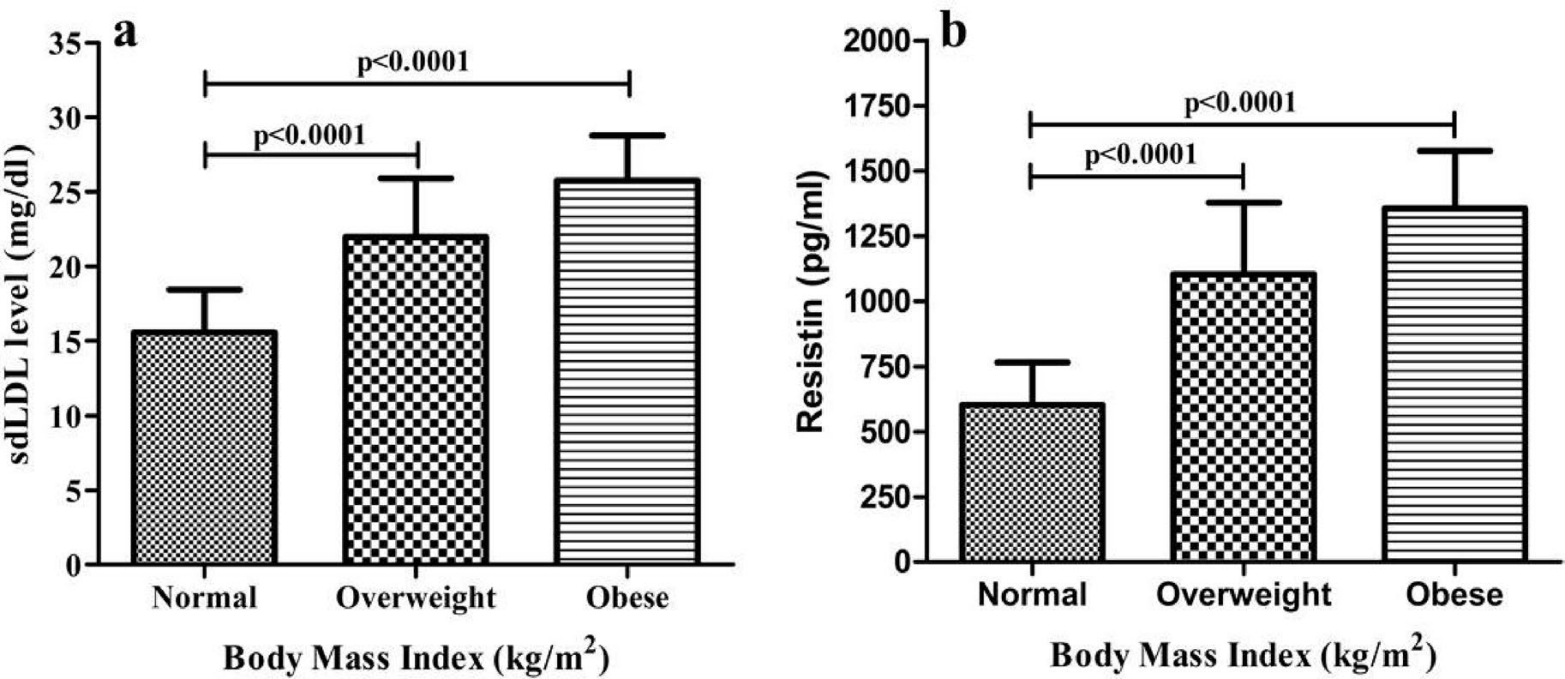
Body mass index, sdLDL and resistin level (**a**) sdLDL with different BMI (**b**) Resistin with different BMI.

In the present study, sdLDL (Fig. 2a) and resistin (Fig. 2b) levels were compared across participants with different BMI categories. Mean sdLDL concentrations were 15.55 ± 2.89 mg/dl in the normal BMI group, significantly higher in the overweight group (21.95 ± 3.95 mg/dl, *p* < 0.0001), and highest in the obese group (25.76 ± 3.01 mg/dl, *p* < 0.0001). Similarly, mean resistin levels were 603.5 ± 163.0 pg/mL in normal BMI participants, increasing significantly in overweight individuals (1003.0 ± 274.9 pg/mL, *p* < 0.0001) and reaching the highest values in the obese group (1355.0 ± 220.8 pg/mL, *p* < 0.0001).

### Association of SdLDL with resistin

**Fig. 3:**
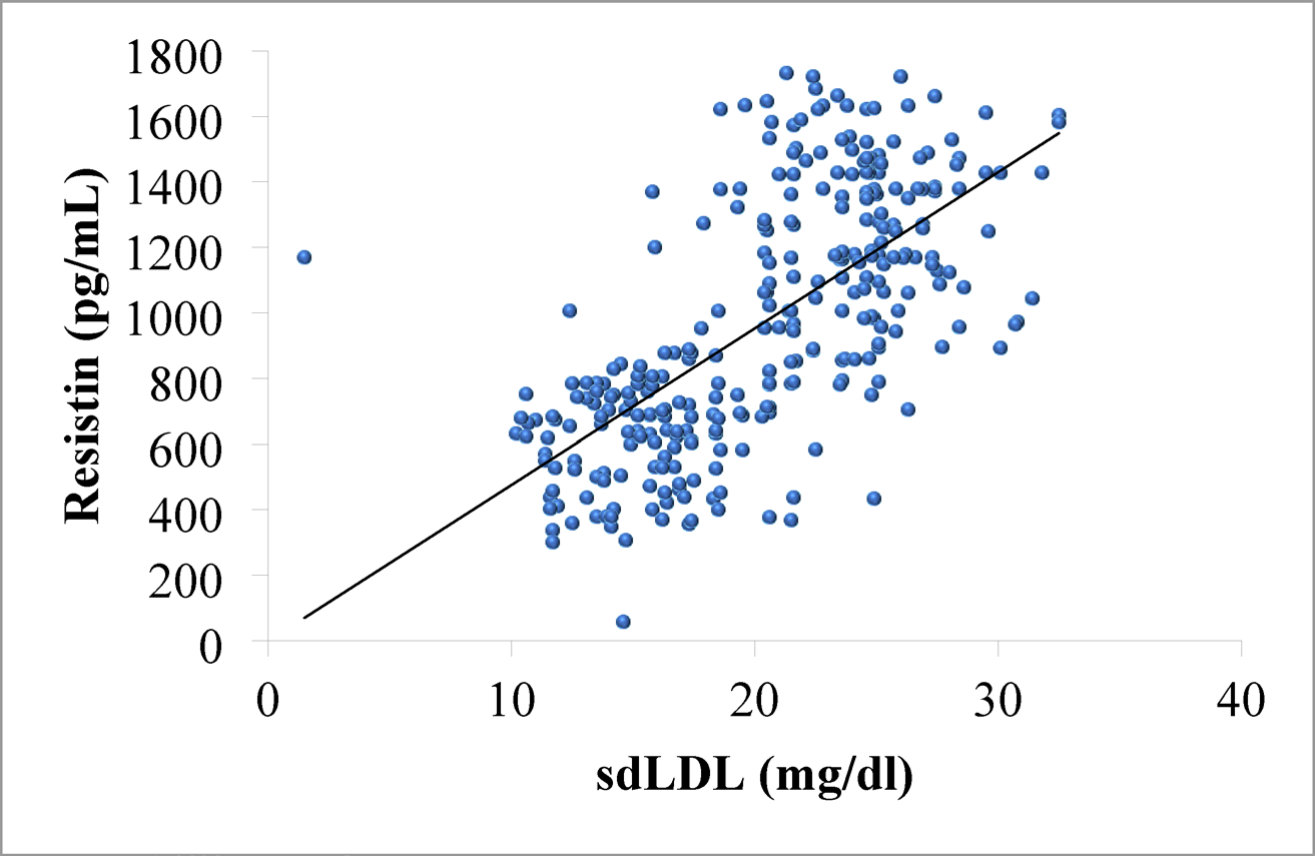
Correlation of sdLDL with resistin level among the study participants.

The association between study participants’ levels of resistin and sdLDL was investigated using correlation analysis (Fig. 3). People with higher sdLDL levels tended to have proportionately higher resistin levels, according to a statistically significant and strong positive correlation (*r* = 0.69, *p* < 0.0001). This association suggests a possible metabolic pathway that is interconnected, whereby elevated pro-inflammatory adipokine activity may coexist with an increase in atherogenic sdLDL particles. The idea that inflammation and dyslipidemia are closely linked in the pathophysiology of obesity-related cardiometabolic risk is supported by this pattern .

### Association of SdLDL and resistin with clinical parameters

Correlation analysis between sdLDL levels and clinical parameters demonstrated (Table 5) that BMI showed the strongest positive association (*r* = 0.77, *p* < 0.0001), followed by total cholesterol (*r* = 0.56, *p* < 0.0001), LDL cholesterol (*r* = 0.57, *p* < 0.0001), VLDL (*r* = 0.57, *p* < 0.0001), triglycerides (*r* = 0.52, *p* < 0.0001), and systolic blood pressure (*r* = 0.47, *p* < 0.0001). A significant negative correlation was observed with HDL cholesterol (*r* = -0.21, *p* < 0.0001). HbA1c and diastolic blood pressure showed weak, non-significant correlations. Similarly, resistin levels were most strongly correlated with BMI (*r* = 0.81, *p* < 0.0001), followed by total cholesterol (*r* = 0.61, *p* < 0.0001), LDL cholesterol (*r* = 0.63, *p* < 0.0001), triglycerides (*r* = 0.60, *p* < 0.0001), systolic blood pressure (*r* = 0.50, *p* < 0.0001), and VLDL (*r* = 0.55, *p* < 0.0001). A stronger inverse association with HDL cholesterol was observed (*r* = -0.29, *p* < 0.0001). HbA1c demonstrated a weak but significant correlation (*r* = 0.15, *p* = 0.02), whereas diastolic blood pressure was not significantly associated. Overall, both sdLDL and resistin levels exhibited strong positive correlations with obesity indices (BMI), atherogenic lipid fractions (LDL, VLDL, triglycerides, cholesterol), and systolic blood pressure, while showing inverse associations with HDL cholesterol, highlighting their interlinked roles in dyslipidemia and cardiometabolic risk.

**Table 5:** Correlation analysis of SdLDL and resistin with clinical parameters such as BMI, HbA1c, systolic, diastolic, HDL, TG, cholesterol, VLDL, LDL

sdLDL with clinical parameters	Correlation coefficient (*r*)	*P* value	Resistin with clinical parameters	Correlation coefficient (*r*)	*P* value
BMI	0.77	< 0.0001	BMI	0.81	< 0.0001
HbA1c	0.08	0.16	HbA1c	0.15	0.02
Systolic blood pressure	0.47	< 0.0001	Systolic blood pressure	0.50	< 0.0001
Diastolic blood pressure	0.09	0.10	Diastolic blood pressure	0.01	0.26
HDL	-0.21	< 0.0001	HDL	-0.29	< 0.0001
TG	0.52	< 0.0001	TG	0.60	< 0.0001
Cholesterol	0.56	< 0.0001	Cholesterol	0.61	< 0.0001
VLDL	0.57	< 0.0001	VLDL	0.55	< 0.0001
LDL	0.57	< 0.0001	LDL	0.63	< 0.0001

### Association of BMI with sdLDL, resistin, smoking, and hypertension

A multiple logistic regression was performed to identify predictors of body mass index (BMI) category (Table 6). The analysis revealed that sdLDL, resistin, and hypertension were significant independent predictors. Specifically, for each one-unit increase in sdLDL, the odds of being in the higher BMI category increased by 37.3% (OR = 1.373, 95% CI [1.183, 1.593], *p* < 0.0001). Similarly, each one-unit increase in resistin was associated with a 1.5% increase in odds (OR = 1.015, 95% CI [1.009, 1.020], *p* < 0.0001). Hypertension was a strong predictor, with affected individuals having over 7.950 times the odds of being in the higher BMI category compared to those without hypertension (OR = 7.950, 95% CI [2.888, 21.888], *p* < 0.0001). In contrast, smoking status was not found to be a statistically significant predictor in this model (OR = 0.686, 95% CI [0.189, 2.491], *p* = 0.567).

**Table 6:** Association of BMI with other parameters by multivariate logistic regression analysis (BMI as the dependent variable)

BMI as dependent variable	B	S.E.	*P*	Exp(B)	95% CI	
					Lower	Upper
sdLDL	0.317	0.076	< 0.0001	1.373	1.183	1.593
Resistin	0.015	0.003	< 0.0001	1.015	1.009	1.020
Smoking	-0.377	0.658	0.567	0.068	0.189	2.491
Hypertension	2.073	0.517	< 0.0001	7.950	2.888	665.297

A gender-stratified logistic regression analysis was performed for males to identify parameters associated with body mass index (BMI) category. As shown in Table 7, both SdLDL and resistin were significant positive predictors. For each one-unit increase in SdLDL, the odds of males being in the higher BMI category increased by 44.1% (OR = 1.441, 95% CI [1.221, 1.701], *p* < 0.0001). Similarly, each one-unit increase in resistin was associated with a 1.0% increase in odds (OR = 1.010, 95% CI [1.006, 1.014], *p* < 0.0001).

**Table 7:** Gender (male) stratified association of BMI with other parameters by logistic regression analysis (BMI as the dependent variable)

BMI as dependent variable	B	S.E.	Sig.	Exp(B)	95% CI	
					Lower	Upper
sdLDL	0.365	0.085	< 0.0001	1.441	1.221	1.701
Resistin	0.010	0.002	< 0.0001	1.010	1.006	1.014

In the gender-stratified analysis for females, the logistic regression model revealed a distinct pattern of associations. As shown in Table 8, SdLDL was not a statistically significant predictor of BMI category in females (OR = 1.186, 95% CI [0.935, 1.504], *p* = 0.161), this could be due to the fact that fewer female participants were included in study. However, resistin remained a significant positive predictor. For each one-unit increase in resistin, the odds of females being in the higher BMI category increased by 1.1% (OR = 1.011, 95% CI [1.003, 1.019], *p* = 0.008).

**Table 8:** Gender (female) stratified association of BMI with other parameters by logistic regression analysis (BMI as the dependent variable)

BMI as dependent variable	B	S.E.	Sig.	Exp(B)	95% CI	
					Lower	Upper
sdLDL	0.170	0.121	0.161	1.186	0.935	1.504
Resistin	0.011	0.004	0.008	1.011	1.003	1.019

**Fig. 4:**
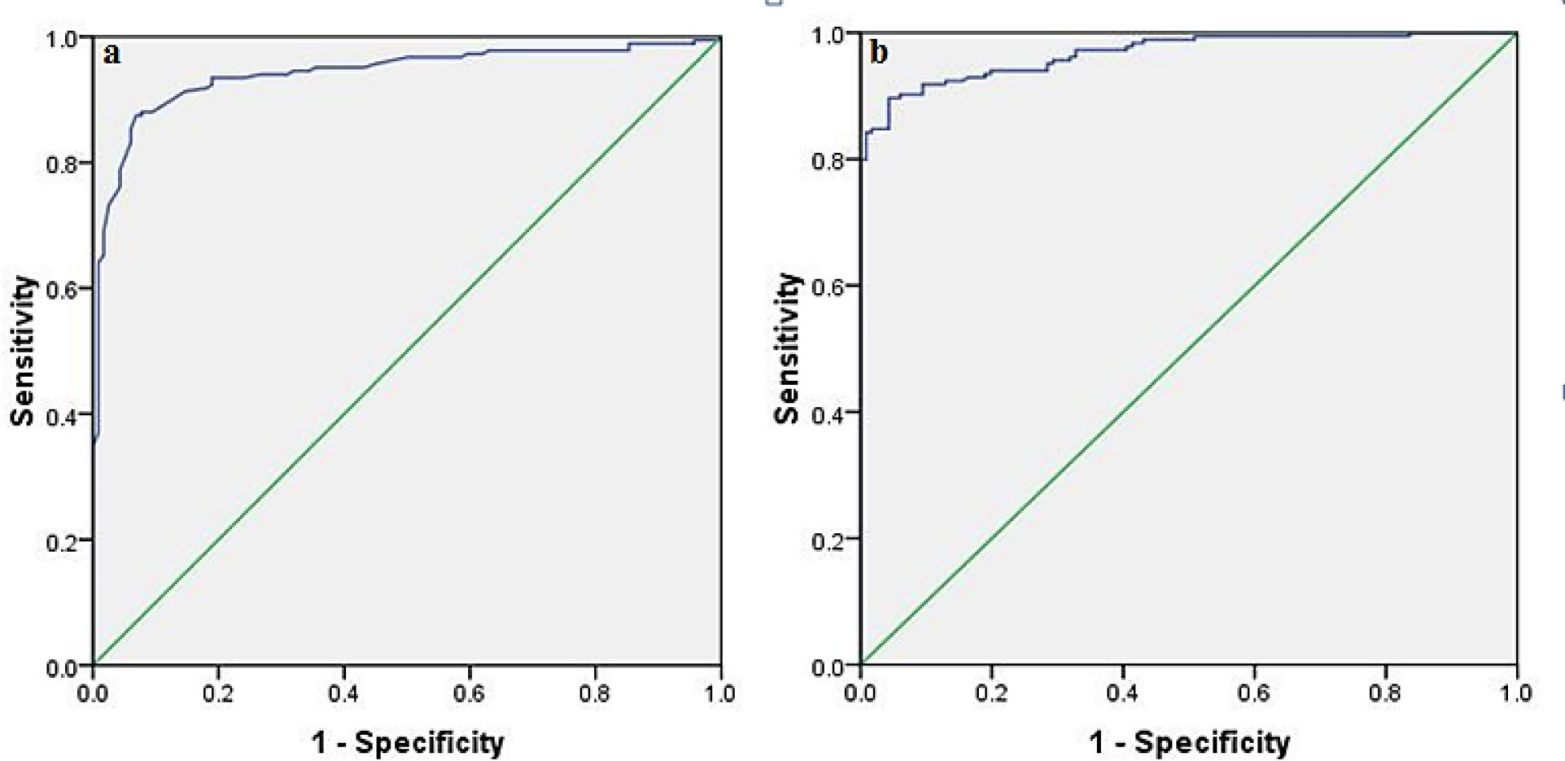
ROC for normal BMI vs. overweight and obesity (**a**) sdLDL for normal BMI vs. overweight and obesity (**b**) resistin for normal BMI vs. overweight and obesity.

**Prognostic significance of SdLDL and resistin**: receiver operating characteristic (ROC) curve analysis was performed to evaluate the prognostic performance of SdLDL and resistin in discriminating the study outcome (Table 9; Fig. 4a and b). SdLDL demonstrated an area under the curve (AUC) of 0.94 (95% CI: 0.91–0.96, *p* < 0.0001), with an optimal cutoff value of 18.55 mg/dl yielding an accuracy of 89%, precision of 90.8%, sensitivity of 90%, and specificity of 88%. Resistin showed a slightly higher AUC of 0.96 (95% CI: 0.95–0.98, *p* < 0.0001), with an optimal cutoff of 750 pg/mL, achieving an accuracy of 88.3%, precision of 88.2%, sensitivity of 93%, and specificity of 81%. These findings indicate that both SdLDL and resistin are strong predictors, with resistin showing marginally superior predictive ability. SdLDL and resistin May have the potential role in predicting cardiometabolic complications (e.g., insulin resistance, dyslipidemia, hypertension).

**Table 9:** ROC curve for SdLDL and resistin for their prognostic efficacy.

Parameters	AUC (95% CI)	Accuracy	Precision	Sensitivity	Specificity	Cutoff	*P* value
sdLDL	0.94 (0.91–0.96)	89%	90.8%	90%	88%	18.55 mg/dl	< 0.0001
Resistin	0.96 (0.95–0.98)	88.3%	88.2%	93%	81%	750 pg/mL	< 0.0001

## Discussion

Substantial increase in global obesity, which has led to an increase in human morbidity and death. Morbid obesity is considered a serious health risk^21^. As previously noted that metabolic consequences of obesity in people are linked to higher risks for a number of illnesses, including insulin resistance, cardiovascular disease, hypertension, and several types of cancer^22^. SdLDL concentration is connected with a higher susceptibility for endothelial penetration and subsequent atherogenesis^23^ and several biomarkers, including elevated blood TG, TC, and LDL, are linked to elevated sdLDL^24^. High sdLDL levels are influenced by a number of illnesses, including obesity^23^, MetS^12^, , systemic hypertension^25^, and hepatic conditions^26^.

Present research compared the biochemical parameters between normal BMI, overweight and obesity, and observed higher HbA1c, LDL, TG, Cholesterol, VLDL among obese and overweight participants while lower HDL level among obese and overweight participants compared to normal BMI. Notably, higher sdLDL were observed among obese participants (BMI: ≥30 kg/m2) compared to overweight (BMI: 25–29.9 kg/m2) and those with normal BMI (BMI: 18.5–24.9 kg/m2).Similarly elevated sdLDL were observed in participants with hypertension and smokers relatively to their counterparts. SdLDL, a particularly type of cholesterol, was highest in obese individuals and also elevated in those with hypertension and smokers suggested that sdLDL as a critical biomarker linked to both obesity and other major cardiovascular risk factors. Similarly, Nakamura M et al. (2021) elevated sdLDL was reported in cigarette smokers and those who quit smoking for last 5 years had significantly lower sdLDL level^27^ and sdLDL shown to be elevated in obese participants found to be linked with atherogenic potential and connected with higher risk of atherosclerotic cardiovascular diseases^23^. Substantial changes in sdLDL across the normal BMI, overweight and obese category with higher sdLDL among the obese participants^28^. Previous studies reported higher serum sdLDL among obese participants’ vs. normal weight participants^29^ similarly in a research by Nikolic D el at. (2013) highlight that higher sdLDL, TG, VLDL and low HDL contribute to increased risk for cardiovascular as well as in development of metabolic syndrome suggested that the therapeutic target to sdLDL could mitigate the lowering risk of cardiovascular disease among the obese people^3^ as well as it has been revealed that the people with higher sdLDL had three to seven times higher risk of developing coronary heart disease^30^. Fan et al. investigated the interconnection between sdLDL and metabolic syndrome in cross sectional study involving Chinese participants revealed that the people with higher sdLDL level had 8.39 fold greater odds of having metabolic syndrome as well as traditional risk factor for obesity and systemic inflammation^30^. Sirikul K et al. (2015) also found higher sdLDL concentration in obease participants compared to normal-weight participants^31^. Study by Bamba V et al. (2007) refer that the weight gain in linked with higher TG, LDL and sdLDL with low HDL has been linked with atherogenic lipid profile^32^. Collectively all the studies emphasize the critical role of sdLDL as biomarker for cardio metabolic risk in obese people. Similarly, in present study, we also observed elevated TG, LDL and sdLDL among the obese participants.

In our study obese (BMI ≥ 30 kg/m²) and overweight (BMI 25–29.9 kg/m²) participants had higher resistin level compared to its counterpart, findings that supports the role of resistin as a biochemical adipokine actively involved in and associated with the pathophysiology of obesity. The pathophysiology of obesity has been connected to the inflammatory cytokine resistin, which is mostly generated by immune cells and adipose tissue. It is also associated with systemic inflammation, insulin resistance, and atherosclerosis^33,34^. Higher resistin level was found in the cardiac dysfunction such as cardiac failure, left ventricular dysfunction and CVD^35^. Study observed higher resistin level among the obese participants compared to normal weight participants^19^ as well as obese diabetic females had higher resistin level relatively overweight and lean participants^36^ suggesting to be risk factor of diabetes in obese people. A study on human subjects showed elevated resistin level in obese subject compared to lean individuals, suggesting potential link between resistin and obesity^37^. The metabolic and secretory profile of various tissues has benn connected with resistin level in human^38,39^. Elevated blood resistin levels have been implicated in athogenesis of insulin resistence, T2DM, atherosclerosis, and cardiovascular diseases in both animals and humans^40^. Elevated serum resistin level in obese individuals compared to normal BMI were positively correlated with TG and inversely correlated with HDL level^41^ which is consistent with the present study findings. Hypertensive obese participants exhibit significantly elevated srum resistin level, suggesting a possible connection between resistin and hypertension pathophysiology of hypertension^42^. Higher resistin level associated with smoking suggested that the smoking can influence the resis level can be contributory factor in metabolic alteration in smokers^43^. Obesity, small dense LDL-cholesterol, and resistin interact in a complex web of physical dysfunctions that are greatly influenced by the interrelated roles of aging, gender, and other illnesses. Age-related metabolic process deterioration, including muscle loss, decreased pancreatic beta cell function, and increased abdominal fat, exacerbates the body’s resistance to insulin. This resistance is the primary cause of unhealthy blood fat levels that encourage artery hardening, which is characterized by a high concentration of small, dense LDL particles resulting from increased activity of hepatic lipase and cholesteryl ester transfer protein^44,45^. Further perpetuating metabolic dysfunction, this aging-associated insulin resistance and inflammation also triggers the release of pro-inflammatory adipokines, particularly resistin, by adipocytes and macrophages^46,47^. Because male androgen patterns promote the accumulation of abdominal fat and lower adiponectin levels, gender plays a crucial role in diversity. Compared to women before menopause, this leads to a lipid profile that is more likely to cause atherosclerosis, as evidenced by higher levels of sdLDL^48,49^. As people age, their metabolic problems associated with being overweight worsen because mitochondria’s capacity to function properly in fat tissue decreases more quickly and cell degradation is encouraged. This results in an inflammatory condition called inflammaging, which directly damages the lining of blood vessels and the pancreatic β-cells^50^. Compared to women, men are more likely to have belly fat because of the influence of androgens, which is linked to increased production of inflammatory substances and decreased adiponectin. This results in a lipid profile that promotes plaque buildup in arteries and an increased risk of heart-related illnesses^51^. In menopause women, when estrogen’s protective effects end, quickly transition to a body composition that is dominated by visceral fat. Their vulnerability to coronary artery disease and type 2 diabetes is significantly increased by this alteration, reaching levels that are comparable to or higher than those seen in men of comparable age^52^.

Present research work revealed higher resistin level in hypertensive participants to its counterpart as well as in smokers. These results revealed that the role of resistin in involvement in metabolic changes among the hypertensive and smokers. Positive correlation between sdLDL with resistin levels were observed among the obese participant suggesting that the increase in sdLDL could contribute in elevation of resistin concentrations. SdLDL and resistin level observed to be positively connected with BMI, HbA1c, systolic and diastolic blood pressure, triglycerides (TG), total cholesterol, VLDL, and LDL levels, while showed negative association with HDL level suggesting potential contributory factor for dyslipidemia.

The current study clarifies that the smoking was not a significant factor in our multivariate logistic regression analysis, but sdLDL, resistin, and hypertension were found to be strong, independent predictors of a higher BMI category. It is noteworthy that hypertension showed a particularly strong correlation with obesity, as evidenced by an odds ratio greater than 83. Obesity is known to contribute to hypertension through mechanisms such as renin-angiotensin-aldosterone system activation, increased sympathetic nervous system activity, and salt retention^53^. The strength of this association in our investigation highlights the important clinical relationship between hypertension and obesity. Notably, our findings demonstrated an important association of resistin and sdLDL with BMI. The positive correlation between sdLDL and BMI supports the concept of obesity-induced dyslipidemia, which is characterized by both quantitative and qualitative changes toward more atherogenic, small, dense LDL particles^54^. Its association with resistin further supports its function as a pro-inflammatory adipokine that links insulin resistance to excess adipose tissue^55^. One of the key observations was the gender-specific connection between sdLDL and BMI, which was significant for men but not for women. This sexual dimorphism may be explained by the positive effects of estrogen on lipid metabolism, including controlling the size of LDL particles^56^. Alternatively, a smaller sample size for females would have decreased the statistical power to detect a significant effect, therefore, more female subjects could be included in future studies to increase the statistical power to make a conclusion about female participants. However, resistin remained a strong predictor in both sexes, suggesting that its association with obesity may be a more universal mechanism that is less affected by sex hormones. While smoking is not importantly associated with weight control, population-based research has shown complex and often contradictory associations between smoking, weight, and metabolic syndrome, which may account for smoking’s lack of independent predictive ability in this specific model^57^. Both sdLDL and resistin are significant biochemical causes of obesity, with sdLDL having a stronger effect in men. The close association with hypertension emphasizes how important it is to control both blood pressure and weight. These results suggest that a gender-specific approach might be beneficial for treatment and prevention strategies, particularly those that focus on lipid profiles. Prognostic significance was also calculated and revealed that the sdLDL and resistin level could be the indicator to predict cardiometabolic complications (e.g., insulin resistance, dyslipidemia and hypertension) at the cutoff value of 18.55 mg/dl for sdLDL and 750pg/mL for resistin which could help to minimize the risk of developing related comorbidities. The results are consistent with resistin’s known function as a major inflammatory adipokine implicated in metabolic dysregulation^55^.

## Conclusion

In conclusion compared to the overweight and normal BMI groups, obese people had significantly higher levels of HbA1c, LDL, triglycerides, cholesterol, VLDL, sdLDL, and resistin, while HDL levels were lower. Resistin and sdLDL had an inverse relationship with HDL and a positive correlation with BMI, systolic blood pressure, and atherogenic lipid parameters. Resistin and hypertension are strong, universal predictors of a higher BMI category. However, the association between sdLDL and BMI is gender specific, being a significant risk factor only in males, however large sample size of data is required to conclude it. Based on ROC analysis, resistin and sdLDL can be used as an indicator for the assessment of overweight and obesity. These results demonstrate how pro-inflammatory adipokines and dyslipidemia are linked to metabolic risk associated with obesity as well as in cardiovascular risk assessment and early intervention strategies.

## Limitations of study

 The cross-sectional design, lack of age and sex matching, small sample size, and unmeasured confounders like dietary practice, and physical activity (duration and intensity),  might have an impact on the associations found in this study. As well as lack of data on medication, such as the uses of lipid-lowering or antihypertensive drugs, which could significantly affect sdLDL and resistin levels. To validate these results, larger longitudinal studies involving balanced cohorts (including males and females in a 1:1 ratio) and thorough lifestyle data are required.

## Data Availability

On reasonable requests data could be available by the corresponding author. We confirm that the data used during the research will not be shared with anybody/broadcasted in any public domain. The datasets generated during and/or analysed during the current study are available from the corresponding author on reasonable request.
